# Establishing disability weights for congenital pediatric surgical conditions: a multi-modal approach

**DOI:** 10.1186/s12963-017-0125-5

**Published:** 2017-03-04

**Authors:** D. Poenaru, J. Pemberton, C. Frankfurter, B. H. Cameron, E. Stolk

**Affiliations:** 1 McMaster Pediatric Surgery Research Collaborative, Dept. of Surgery, McMaster University, Hamilton, Canada; 2 MyungSung Medical College, Addis Ababa, Ethiopia; 3 Department of Surgery, Montreal Children’s Hospital, McGill University, 16-4200 Sherbrooke West, Westmount QC H3Z 1C4, Montreal, Canada; 4 Dept. of Clinical Epidemiology and Biostatistics, McMaster University, Hamilton, Canada; 5 Institute for Medical Technology Assessment, Erasmus Medical Center Rotterdam, Rotterdam, The Netherlands

## Abstract

**Background:**

Burden of disease (BoD) as measured by Disability-Adjusted Life Years (DALYs) is one of the criteria for priority-setting in health care resource allocation. DALYs incorporate disability weights (DWs), which are currently expert-derived estimates or non-existent for most pediatric surgical conditions. The objective of this study is to establish DWs for a subset of key pediatric congenital anomalies using a range of health valuation metrics with caregivers in both high- and low-resource settings.

**Method:**

We described 15 health states to health professionals (physicians, nurses, social workers, and therapists) and community caregivers in Kenya and Canada. The health states summaries were expert- and community-derived, consisting of a narrated description of the disease and a functional profile described in EQ-5D-5 L style. DWs for each health state were elicited using four health valuation exercises (preference ranking, visual analogue scale (VAS), paired comparison (PC), and time trade-off (TTO)). The PC data were anchored internally to the TTO and externally to existing data to yield DWs for each health state on a scale from 0 (health) to 1 (dead). Any differences in DWs between the two countries were analyzed.

**Results:**

In total, 154 participants, matched by profession, were recruited from Kijabe, Kenya (*n* = 78) and Hamilton, Canada (*n* = 76). Overall calculated DWs for 15 health states ranged from 0.13 to 0.77, with little difference between countries (intra-class coefficient 0.97). However, DWs generated in Kenya for severe hypospadias and undescended testes were higher than Canadian-derived DWs (*p* = 0.04 and *p* < 0.003, respectively).

**Conclusions:**

We have derived country-specific DWs for pediatric congenital anomalies using several low-cost methods and inter-professional and community caregivers. The TTO-anchored PC method appears best suited for future use. The majority of DWs do not appear to differ significantly between the two cultural contexts and could be used to inform further work of estimating the burden of global pediatric surgical disease. Care should be taken in comparing the DWs obtained in the current study to the existent list of DWs because methodological differences may impact on their compatibility.

**Electronic supplementary material:**

The online version of this article (doi:10.1186/s12963-017-0125-5) contains supplementary material, which is available to authorized users.

## Background

The global health data provided through the Global Burden of Disease (GBD) study [1] using the Disability-Adjusted Life Year (DALY) metric has been a key component in the development of health policy, especially in low- and middle-income countries (LMICs). In such settings, in the absence of available primary data, GBD data have been proposed and used for broad health care initiatives, such as the Lancet Commission on Global Surgery [2]. Within specialty areas, this necessitates a level of granularity that has not been originally intended or provided. Such is the case of pediatric surgery, where concerted efforts to improve access to care and quality of care lack data support.

In 2006, Debas conservatively estimated that 11% of the GBD was being attributed to “surgical disease,” [3] i.e., health conditions primarily treated through surgical intervention. More recently, Shrime et al. placed this percentage to as high as 30% [4], though the methodology used to derive this figure is not clear. Disproportionately carrying this surgical burden are children in LMICs, who have garnered increased attention in the global surgery community [5]. Congenital anomalies, one of the largest subsets of pediatric surgical conditions, are believed to account for 1.9% of the GBD [6], although this is likely to be an underestimate due to the limited number of conditions studied and the difficulty associated with capturing buden of disease (BoD) data [7]. The primary objective of this study was thus to enable the estimation of DALYs for a subset of key pediatric congenital anomalies.

The DALY is a widely used metric in LMICs, developed to quantify BoD and inform global priority-setting and resource allocation [8, 9]. It encompasses both mortality and morbidity by combining the number of years lost due to premature mortality (Years of Life Lost, YLLs) with the Years Lived with Disability (YLDs). Calculating the latter requires a disease-specific disability weight (DW), which is an empirically determined factor reflecting the health decline associated with each health state, ranging between 0 (perfect health) and 1 (death) [7, 10]. Estimation methodologies for DWs are wide-ranging and potentially contentious [11]. All valuation methods are by definition judgmental tasks solved by participants at the moment of the exercise, and compatibility data are mixed as best [8]. There is no reason to expect the same results across all methods, yet comparability with other DWs remains a key requirement in the GBD context to prevent methodological differences impacting the outcomes of BoD comparisons across countries and disease areas. A preferred option therefore is to construct new DWs using similar estimation methods and assumptions as used in the GBD context, although this has been somewhat of a moving target.

Many different methods for DW estimation have developed over time. DWs may for instance be elicited through various psychometric exercises [12] or by trade-off methods [13]. The former include ranking exercises, magnitude estimation, visual analogue scaling (VAS), and pairwise comparison (PC) or rank ordering tasks. The latter comprise the standard gamble, time trade-off (TTO), and person trade-off (PTO) [8] methods. The earliest DALY version appeared in a 1993 World Development Report, assigning conditions to various degrees of perceived disability [14]. In the second DALY version by Murray and Lopez, published in 1996 as part of the GBD 1990 study [15], medical expert decision-makers valued a subset of 22 indicator disease-oriented scenarios using the PTO method, then used the rating scale generated for the entire set of 131 conditions. The Dutch Disability Weights Project [16] expanded the available weights by eliciting PTO values for another set of conditions described using the EQ-5D and an additional cognition dimension [17]. Subsequent modeling of those Dutch data by the Australian BoD team further expanded the set of available DWs [18]. In the most recent GBD update [19], the methodology was significantly changed to a world-wide survey of over 30,000 household- and web-based PCs covering 220 unique health states. The results of the PCs were then anchored on a subset of 30 health states for which population health equivalence choices were elicited through one of the four used web-based surveys. Other parallel efforts in North America include the US National Institutes of Health DALY study [8], and the Public Health Agency of Canada’s Classification and Measurement System of Functional Health (the CLAMES system) [20]. Haagsma et al. offers a comprehensive review of DW methods and studies published through 2012 [21].

The methods have clearly advanced with the scope of DW investigation. The methods used in the most recent GBD update are flexible and generate a high level of granularity by adopting the PC method while minimizing complexity of respondents’ task through a limited number of complex population health equivalence choices [19]. Despite the above efforts, DW values for many surgical conditions, particularly within subspecialties, are missing [22], thus rendering the quantification of surgical BoD challenging. Moreover, the original and subsequent GBD studies have summarized health states and their sequelae by age groups, regions and countries, rather than analyzing them by (sub) specialty. As a result, the burden of surgical conditions affecting children, especially in LMICs, has not been formally estimated, and their DWs are conspicuously missing [3]. In fact the 2006 extensive volume on the GBD study only included DW values for seven congenital surgical conditions in four disciplines, themselves pulled from the original GBD 1990 study [7], and there were none in GBD 2010. In their absence, surgical specialty literature has used DW proxies, estimated by expert opinion using ballpark disability descriptions [23–26].

This study intends to address the above gaps by investigating DWs for 15 congenital pediatric surgical conditions. Given the controversy surrounding the influence of cultural factors on the DW process [26], this study’s DWs were derived in both Canada and Kenya. In developing our strategy we acknowledged the GBD study viewpoint that achieving comparability of DWs across countries, time periods, and – in our case – disease areas is of utmost importance. While the possibilities to achieve this perfectly are inherently limited because data will necessarily be collected at a different moment in time and in different resource contexts, we attempted to broaden our methodology while maintaining as high a comparability of assumptions and methods with the original data estimates as possible.

## Methods

### Study design and participants

Data were collected for this study in Kijabe, Kenya and Hamilton, Canada between March and August 2012. Research ethics approval was obtained at both institutions (AIC Kijabe Hospital and Hamilton Integrated Research Ethics Board [11–328]) and written consent obtained from all participants. Total sample size was based on feasibility of recruitment at both centers.

Focus groups at both sites were conducted primarily in English, with Kenyan community groups conducted in Swahili and then translated. Participants were selected based on experience with pediatric congenital anomalies (balancing experienced and non-experienced) and were recruited to match roles (i.e., physician, nurse, social worker, therapist, community participant) between the two sites. Data were collected in Kenya over 2 weeks at AIC Kijabe Hospital and in a community setting in Nairobi, and in Canada over 3 months at McMaster Children’s Hospital. Each participant completed all study instruments in a single 3-hour session. Focus groups were facilitated by a local research assistant and the research coordinator, and comprised 5–15 participants based on individual availability.

### Health state descriptions

**Fig. 1:**
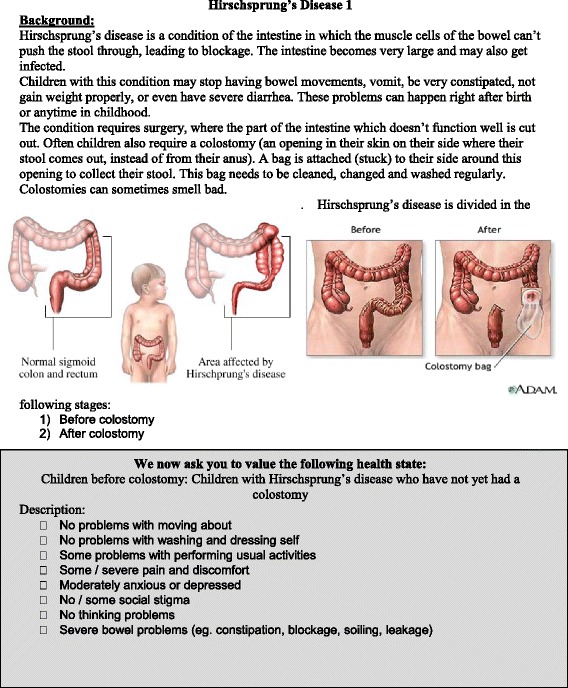
Health state information example

We developed a set of lay descriptive handouts as suggested by Rehm and Frick [8] for each of 15 health states (mild/severe hypospadias, undescended testis, cleft lip, cleft palate, mild/severe imperforate anus, Hirschsprung’s Disease before/after colostomy, mild/severe spina bifida, mild/severe abdominal wall defect, hydrocephalus, and intestinal atresia). An example of a handout is shown in Fig. 1, and all handouts are available online (Additional file 1). These health states were chosen based on a ranking of the most prevalent congenital pediatric surgical conditions encountered at both sites. The handouts were circulated amongst an expert panel for face validation of the lay descriptions of functional health status and symptoms of each state; diagrams were included to improve understanding. Each handout comprised a disability profile description on eight domains, including the five EQ-5D dimensions (mobility, self-care, usual activities, pain, mood) [14], and three additional domains: “cognitive functioning,” “evacuation problems,” and “social stigma”. The three additional domains were informed by the CLAMES study [17], the Dutch Disability Weights project [13], and from our qualitative community-based focus groups with Kenyan caregivers of children with neural tube defects exploring culturally-based social stigma [27] as suggested by Kapiriri et al. [28].

Based on severity and surgical management, some conditions were divided into two distinct health states (e.g., Hirschsprung’s before and after colostomy). All valuations applied only to the health states before definitive treatment (*untreated*) – thus a state such as “Hirschsprung’s after colostomy” referred to a temporary procedure still requiring a definitive surgery. Five health states also had DWs derived by the GBD 1990 study [13] which were used as the gold standard and were compared against our newly derived DWs.

### Valuation tasks

Standard protocols were developed for research staff training and participant explanations. Prior to data collection at each site, a pilot focus group with a representative sample was conducted to assess understanding and language for each exercise and for the lay description handouts using a series of Likert scales.

All participants completed four health valuation exercises for each health state, including preference ranking (PR), visual analogue scales (VAS), paired comparisons (PC), and time trade-off exercises (TTO) [29]. Participants were asked to complete the exercises in the following order: PR, VAS, PC, TTO. The PR task was introduced to familiarize participants with the various health states and obtain an understanding of their relative severity. The VAS task was then initiated to introduce the concept of health valuation and to ensure their understanding of the health state descriptions and the purpose of the exercises. After these simpler tasks, the participants then completed the more complex PC and TTO exercises which were used as the primary data for this study. The PC method was specifically chosen for consistency with GBD methodology. PC data, however, are generated on a latent scale, and these values need anchoring to the full health-dead scale in any of several ways. In our case, we were able to harmonize the results with the GBD scale by using the values for overlapping conditions as anchor points for the PC results. Alternatively, the TTO values might be used to identify how the PC-derived latent values relate to the full health-dead scale as shown by Rowen et al. [30]. The use of TTO additionally enables comparison of our DW values with those published in the wider health-related quality of life (HRQoL) literature where TTO is a preferred valuation method. Having these two options was considered relevant as the feasibility and validity of the option of anchoring the new DWs to existing GBD data depends on the level of congruence across the new and existing DWs.

To complete the PR, each participant was given a set of 15 health state index cards in random order, and asked to rank each from least to most severe. Next, participants completed a VAS using a 100-point line anchored by death and perfect health with 5-point increments demarcated on the line. Participants were instructed to mark an exact point on the line for each health state in terms of severity. Additional instructions included placing similar health states closer together and vice versa. Participants then completed a series of PCs that directly compared each health state to every other one, choosing which state was more severe. This resulted in 105 pairwise comparisons (15 *14/2) for each participant.

In the TTO exercise participants were instructed to trade off years of healthy life for years of life lived in the specific health state, as if they were the parent of a child with the condition, and as if they were trading years off their child’s life. The TTO adopted a time frame (T) of 60 years (derived from WHO standard life expectancy rates), and a smallest tradeable unit of 10 years. For example a participant could choose between living for 60 years in a particular health state or living 10, 20, 30, etc. years in perfect health. The TTO exercise was aimed at determining the number of years *t* in perfect health that would make the two options equally attractive (i.e., the indifference point), so that the value of a life year could be computed as *t*/T.

### Statistical analysis

All participants’ individual responses from each valuation measure were included. DWs were calculated for each health state, by each exercise, for each country. We summarized the data from the PR task by averaging the rank order for each health state and transforming the data to a continuous number between 0 and 1. Of note, these scores reflect how good or bad all health states are relative to the value of the best and the worst state in the set, but not relative to health states that were not included in the choice tasks – e.g., dead and full health. Therefore, these scores cannot be used as DWs. For the VAS, direct measurements from the VAS scale were obtained and averaged amongst participants. In the PC exercise the proportion of the number of times each health state was chosen over its comparator was calculated for each condition, and using the normal curve, the proportions were transformed into Z-scores. The scores associated with each health state were then summed and averaged to yield an overall Z-score corresponding with the probability of a health state being chosen over all others [31]. The resulting score is a DW that is estimated on a latent scale (the resulting values are not yet anchored on the full health-dead scale). Addition of the magnitude of the most negative score and subsequent division by the highest score was applied to all values to yield a set of weights spanning a range of 0 to 1. Finally, DWs were calculated from the TTO exercise with the formula “utility = time in full health/time in disease state.”

The final analytical step involved anchoring of the PC-derived values onto the full health-dead scale. There are several ways to achieve this. Relying solely on data collected in this study, the PC scores obtained on latent scales were anchored to mean TTO values (PC-TTO) through linear regression, as suggested by Stouthard et al. [16] and Rowen et al. [30]. Alternatively, the PC-derived values may be anchored on the full health-dead scale by using previously reported DWs, i.e., those from GBD 1990, for the five health states included in both datasets. The Intraclass Correlation Coefficient (ICC) assuming a one-way random model for average measures was used to analyze the agreement between the PC-derived values obtained in our study and the TTO and GBD values.

Formal quantitative data comparisons between sites were analyzed using SPSS v20.0 with a 5% significance level and Z scores computed in an Excel® spreadsheet. Results were presented using summative descriptive statistics with means, standard deviations, and 95% confidence intervals where appropriate. Differences between groups were assessed using either the Fisher Exact Test or the Mann Whitney *U* test, depending on normalcy of the data. All DW data were first explored graphically for trends at each site, as well as descriptively between sites.

## Results

**Fig. 2:**
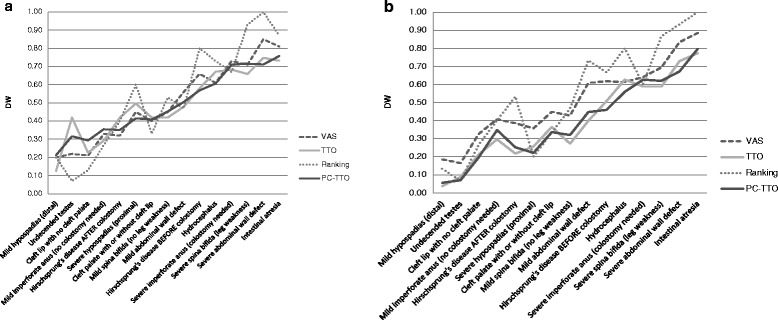
**a** Kenyan Disability Weights per exercise. **b** Canadian Disability Weights per exercise. DW = disability weight; VAS = visual analog scale; TTO = time trade-off method; PC-TTO = TTO-anchored paired comparisons method

In total 154 participants were recruited; 78 from Kenya and 76 from Canada (Table 1). DWs obtained from each of the four exercises (using internally derived PC-TTO values) is depicted in Fig. 2a and b for Kenya and Canada, respectively.

**Table 1:** Study participant characteristics (*n* = 154)

Participants	Kenya	Canada
*Healthcare professionals*	*60*	*64*
Physicians	20	25
Nurses	35	32
Allied health professionals	5	7
Community members	18	12
Total	78	76

Tables 2 and 3 detail the DW values obtained at each site by all methods, including both internal (TTO) and external (GBD) anchoring of PC values.

**Table 2:** Kenyan disability weights per tool (*n* = 78)

Health state	Ranking	VAS	TTO	PC-TTO	PC-GBD
Mild hypospadias	0.2	0.2	0.126	0.212	0
Undescended testes	0.07	0.22	0.419	0.317	0.115
Cleft lip with no cleft palate	0.13	0.21	0.226	0.293	0.076
Mild imperforate anus	0.27	0.33	0.293	0.356	0.179
Hirschsprung’s disease AFTER colostomy	0.4	0.32	0.419	0.351	0.170
Severe hypospadias	0.6	0.45	0.496	0.415	0.275
Cleft palate with/without cleft lip	0.33	0.4	0.419	0.408	0.264
Mild spina bifida	0.53	0.45	0.419	0.452	0.336
Mild abdominal wall defect	0.47	0.56	0.479	0.505	0.421
Hirschsprung’s disease BEFORE colostomy	0.8	0.66	0.581	0.569	0.526
Hydrocephalus	0.73	0.61	0.671	0.607	0.587
Severe imperforate anus	0.67	0.73	0.683	0.710	0.756
Severe spina bifida	0.93	0.71	0.659	0.716	0.765
Severe abdominal wall defect	1	0.85	0.748	0.711	0.758
Intestinal atresia	0.87	0.81	0.732	0.758	0.834

*VAS* visual analogue scale, *TTO* time trade-off method, *X-rank* TTO/VAS anchored ranking method, *X-PC* TTO/VAS anchored paired comparisons method

**Table 3:** Canadian disability weights per tool (*n* = 78)

Health state	Ranking	VAS	TTO	PC-TTO	PC-GBD
Mild hypospadias (distal)	0.133	0.185	0.037	0.055	0
Undescended testes	0.067	0.164	0.087	0.069	0
Cleft lip with no cleft palate	0.267	0.326	0.213	0.196	0.058
Mild imperforate anus	0.4	0.407	0.296	0.348	0.298
Hirschsprung’s disease AFTER colostomy	0.533	0.385	0.217	0.252	0.146
Severe hypospadias (proximal)	0.2	0.357	0.257	0.221	0.098
Cleft palate with or without cleft lip	0.333	0.448	0.365	0.336	0.280
Mild spina bifida	0.467	0.428	0.272	0.320	0.254
Mild abdominal wall defect	0.733	0.608	0.398	0.447	0.455
Hirschsprung’s disease BEFORE colostomy	0.667	0.618	0.508	0.460	0.475
Hydrocephalus	0.8	0.613	0.627	0.559	0.632
Severe imperforate anus	0.6	0.641	0.589	0.627	0.740
Severe spina bifida	0.867	0.694	0.587	0.620	0.729
Severe abdominal wall defect	0.933	0.834	0.729	0.672	0.812
Intestinal atresia	1	0.885	0.773	0.797	1

*VAS* visual analogue scale, *TTO* time trade-off method, *X-rank* TTO/VAS anchored ranking method, *X-PC* TTO/VAS anchored paired comparisons method

**Fig. 3:**
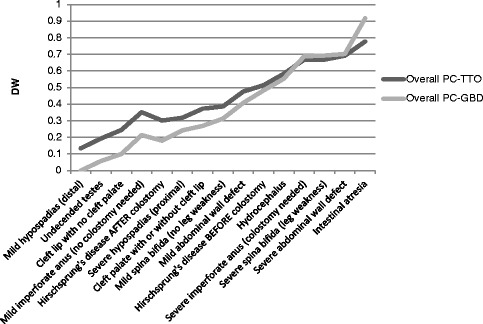
Disability Weights by internal and external anchoring methods. DW = disability weight; PC-TTO = TTO-anchored paired comparisons method; PC-GBD = GBD-anchored paired comparisons method

Comparison of results across the two sites is shown using both TTO-anchored and GBD-anchored PC values in Table 4, and overall values obtained by the two anchoring methods are depicted in Fig. 3.

**Table 4:** Multi-national disability weight comparison

Health state	KE PC-TTO	CAN PC-TTO	KE PC-GBD	CAN PC-GBD
Mild hypospadias (distal)	0.212	0.055	0	0
Undescended testes	0.317	0.069	0.115	0
Cleft lip with no cleft palate	0.292	0.196	0.076	0.123
Mild imperforate anus	0.356	0.348	0.179	0.249
Hirschsprung’s disease AFTER colostomy	0.351	0.252	0.170	0.189
Severe hypospadias (proximal)	0.415	0.221	0.275	0.209
Cleft palate with or without cleft lip	0.408	0.336	0.264	0.273
Mild spina bifida	0.452	0.320	0.336	0.289
Mild abdominal wall defect	0.505	0.447	0.421	0.394
Hirschsprung’s disease BEFORE colostomy	0.569	0.459	0.526	0.439
Hydrocephalus	0.607	0.559	0.5870	0.520
Severe imperforate anus	0.710	0.627	0.7555	0.621
Severe spina bifida	0.716	0.619	0.765	0.620
Severe abdominal wall defect	0.711	0.672	0.758	0.648
Intestinal atresia	0.758	0.797	0.834	1

*DW* disability weight, *SD* standard deviation

In general, discrepancies entailed higher estimated DW values in Kenya, and for severe hypospadias and undescended testes these values were statistically significantly higher than Canadian-derived DWs (*p* = 0.04 and *p* < 0.003, respectively).

**Fig. 4:**
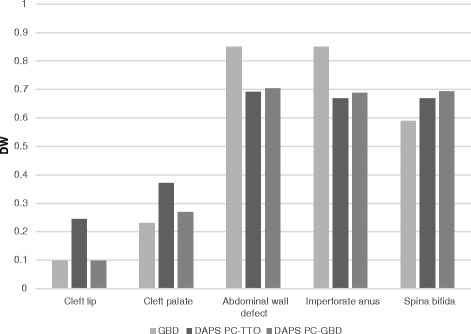
Global Disability Weights for common conditions in GBD 1990 and current study. DW = disability weight; GBD = GBD 1990 study; DAPS = current study; PC-TTO = TTOanchored paired comparisons method; PC-GBD = GBD-anchored paired comparisons method

Disability weights for the common health states included both in our study and the GBD 1990 study are compared in Fig. 4. While values were generally similar for several health states, discrepancies were noted particularly for cleft lip and palate, and these discrepancies were reduced when our PC values were anchored externally to the GBD study.

The ICC showed high levels of reliability between the DW data calculated for both Kenya and Canada (ICC 0.97, 95% CI: 0.93–0.99), as well as between the GBD values, TTO-anchored DW values, and GBD-anchored DW values for the five health conditions common to both studies (ICC = 0.97; 95% CI 0.83–1.0).

## Discussion

The GBD effort over the past two decades has been instrumental in quantifying health burden, needs, and factors both geographically and by broad sets of conditions, thus providing an invaluable body of information to policymakers and health care professionals. The GBD project and its wide adoption by the World Health Organization, World Bank, and several national bodies [32–35] has also been essential in establishing DALYs as the preferred metric globally in BoD measurement. While the GBD project has been extremely comprehensive, its stated global and all-inclusive purpose has resulted in limited granularity within specific medical and surgical specialty areas. In particular in LMICs, in the absence of direct population data, efforts to estimate DALYs are constrained by the available DW values, which are frequently very sparse. The aim of the current study was to generate DW values for a set of congenital pediatric surgical conditions as a way to start filling that gap.

The task was successfully accomplished in both study settings. DW values generated by VAS, ranking, PC, and TTO for the 15 health states spanned the full health-dead spectrum and were generally comparable. Latent scale PC values were alternatively anchored both internally to the TTO and externally to the GBD scale, generating again similar results. With a few exceptions (discussed below), inter-country results showed significant similarity, as documented by the ICC values. Internally generated PC-TTO values correlated well with GBD values for common conditions, and anchoring to these values naturally improved the correlation.

### DW values

In the absence of previous studies within the subspecialty, and faced with a wide choice of valuation methods available, each with its own benefits and shortcomings, the authors chose to start with four different methods, both psychometric and econometric, and compare the results obtained by these broad inputs for each health state. This strategy produced a large number of data points without over-burdening the participants, and allowed inter-method comparisons as well as both a priori and *post-hoc* suggestions for preferred methods. Yet, we also faced the complex question of how to deal with potential discrepancies across the methods. Variation in DW estimates could result from participants’ different health states interpretation, their risk aversion, and time preference, but also from differences in valuations between exercises for the same health state, and overall distributional concerns such as VAS distortion [36].

An anticipated strength of the chosen set of methods was its ability to generate different types of data: PC values being derived on a latent scale can complement other methods while avoiding potential conflicts of scale when other methods are paired (e.g., VAS and TTO). This strategy is increasingly popular [31, 36, 37]. The adoption of PC in the recent version of the GBD study also strongly mitigates in favor of its use on the latent scale. Pooling of values obtained across the other methods has to the best of our knowledge not been done – instead a choice for either one is made based on pros and cons of each. Similarly, while rank data could be used to provide values on a latent scale like PC, the latter is favored for its greater reliability, with rank data often used just as a “warm-up” exercise [38]. Nevertheless, the presence of values derived from multiple methods remains beneficial, allowing at least to assess convergence across methods and demonstrate validity. Against that background we were pleased with the high level of agreement, as shown in the high ICC values, that was achieved across the methods.

The DW values generated for the 15 health states across the two sites were generally similar based on high ICC values, leading support to the assertion that DWs are stable cross-nationally and cross-culturally [16, 39]. Two notable exceptions to this purported DW stability were encountered in the current study. Severe (i.e., proximal) hypospadias and undescended testes were assigned higher DW values in Kenya. This discrepancy may be explained culturally: both health states include the possibility of infertility in their descriptions, a state associated with significant stigma in many non-Western cultures [40, 41].

Limited possibilities exist for external validation of the DW estimates generated in this study. The GBD 1990 study, already used in our study for external anchoring, included DWs for seven congenital surgical conditions (cleft lip, cleft palate, abdominal wall defects, imperforate anus, cardiac defects, esophageal atresia, and spina bifida) [7], and later DW studies globally did not expand this list. Moreover, only the cleft lip and palate states include both untreated and treated values, a significant limitation to the use of other published DW values in surgical arenas. Within the limitation of slightly different methodologies used, the current study had the dual opportunity of using the DW values of the common health states for both external validation (of PC-TTO values) and external anchoring (as in PC-GBD values). We consider the PC-TTO as our primary “take-home” results as they are internally derived and not dependent on overlapping health states with other studies. Moreover, the two methods generally generated similar DW values, well within the same order of magnitude. Of note however, cleft lip and palate received significantly lower values in the GBD study. This may be due to disability from cleft lip/palate being artificially limited to the first 5 years of life in the GBD study, a constraint not reflecting the reality of older children living with this untreated condition in LMICs [42].

### Comparison to GBD 2010 study and advantages

With the recent publication of the GBD 2010 study, any parallel attempt at deriving DWs must be justifiable, valid, and comparable. The primary justification for the current study is simply the necessity to obtain a wider set of DW values within a given specialty, for the purpose of generating relevant specialty-specific BoD data that can inform policy decision-making in this area. Yet in order to offer valid inter-specialty comparisons, the methodology of such parallel studies must be sufficiently similar to that of the GBD gold standard. Without the benefit of the latest iteration of the GBD study at the time of study design, and using a much smaller study sample, the authors chose a panel of valuation methods which allowed the comparison of commonly-used methods in the literature. In light of the current results, the use of paired comparisons appears justified and probably sufficient, in conjunction with a method of anchoring the results to the health-dead scale. This process resembles the GBD study in its use of PCs, though differing from it in the anchoring method. Other strengths of the current study include standardized and explicit health state descriptions, and input from both health care workers and families familiar with the conditions investigated. But caveats remain, such as the different approach to describing the health states, and uncertainty whether the same health state DW values will be obtained in PCs if more or less health states are included in the experiment. The checklist for any such future efforts must include clear, consistent health state descriptions and a single psychometric valuation such as PC, anchored firmly to the disability scale. Moreover, studying health states spanning a wide range of severity would facilitate robust data generation.

The main limitation of the study pertains to the underlying concept of the DALY and of the disability weighting which it requires. In the first place, it is extremely difficult to harmonize universal DW values with the widely divergent sociocultural and economic contexts where they are derived [43]. Furthermore, there are multiple controversial value decisions in the computations of DALYs which can significantly impact the ultimate BoD conclusions drawn from them [44, 45], as well as limitations inherent within the specific health valuation exercises themselves. Finally, DALYs seem to underestimate several specialty areas, such as neglected tropical diseases [46] and surgical conditions [18].

## Conclusions

The current study has successfully generated a set of DW values for pediatric congenital anomalies, making these values available for all necessary future studies [47]. The process involved in generating such a limited DW set was relatively straightforward and inexpensive, and, within the confines of the above limitations, the results were robust and comparable to those generated by large global studies. The DWs do not appear to differ significantly across divergent sociocultural contexts and can be used to calculate both the met and the unmet burden of global pediatric surgical disease [48].

While the extensive global and national BoD studies will remain the basis for global policy decisions, the study suggests that DW sets may be expanded and refined within a surgical specialty. While waiting for future studies to show whether other specialties may be equally successful in the process, a cautious, well-guided advance is recommended in this emerging field in order to ultimately generate practical knowledge in global health.

## Additional file

Additional file 1: Health state information handouts. (PDF 648 kb)

## References

[1] GBD 2015 Disease and Injury Incidence and Prevalence Collaborators (2015). Global, regional, and national incidence, prevalence, and years lived with disability for 301 acute and chronic diseases and injuries in 188 countries, 1990–2013: a systematic analysis for the Global Burden of Disease Study 2013. Lancet.

[2] Meara JG, Leather AJM, Hagander L, Alkire BC, Alonso N, Ameh E (2015). Global Surgery 2030: evidence and solutions for achieving health, welfare, and economic development. Lancet.

[3] Debas H, Jamison D (2006). Surgery. Disease control priorities in developing countries.

[4] Shrime MG, Bickler SW, Alkire BC, Mock C (2015). Global burden of surgical disease: an estimation from the provider perspective. Lancet Glob Health.

[5] Ozgediz D, Poenaru D (2012). The burden of pediatric surgical conditions in low and middle income countries: A call to action. J Pediatr Surg.

[6] World Health Organization (WHO). Global Estimates 2014 Summary Tables: DALY by Cause, Age, and Sex for 2000–2012. World Health Organization Jun 2014. Geneva, Switzerland. http://www.who.int/healthinfo/global_burden_disease/en/. Accessed 1 July 2015

[7] Debas H et al. Disease Control Priorities, Third Ed. Volume 1. Essential Surgery. World Bank. 2015 Washington, DC. https://openknowledge.worldbank.org/handle/10986/21568. Accessed 1 July 2015

[8] Murray CJL (1994). Quantifying the burden of disease: the technical basis for disability-adjusted life years. Bull World Health Organ.

[9] Lopez AD, Mathers CD, Ezzati M, Jamison DT, Murray CJL (2006). Global Burden of Disease and Risk Factors. Disease Control Priorities Project.

[10] Rehm J, Frick U (2010). Valuation of health states in the US study to establish disability weights: lessons from the literature. Int J Methods Psych Res.

[11] Mont D (2007). Measuring health and disability. Lancet.

[12] Revicki D, Kaplan R (1993). Relationship between psycho- metric and utility-based approaches to the measurement of health-related quality of life. Qual Life Res.

[13] Dolan P, Culyer A, Newhouse J (2000). The measurement of health-related quality of life for use in resource allocation decisions in health care. Handbook of Health Economics.

[14] World Bank (1993). World development report 1993; investing in health.

[15] Murray CJL, Lopez AD (1996). A comprehensive assessment of mortality and disability from disease, injures and risk factors in 1990 and projected to 2020. Global burden of disease.

[16] Stouthard M, Essink-Bot ML, Bonsel GJ (1997). Disability Weights for Diseases in the Netherlands.

[17] Brooks R, Group EQL (1996). EuroQoL: The current state of play. Health Policy.

[18] Mathers CD, Vos T, Stevenson C (1999). The burden of disease and injury in Australia Summary report.

[19] Salomon J, Vos T, Hogan DR, Gagnon M, Naghavi M, Mokdad A (2012). Common values in assessing health outcomes from disease and injury: disability weights measurement study for the Global Burden of Disease Study 2010. Lancet.

[20] McIntosh CN, Connor Gorber S, Bernier J, Berthelot JM (2007). Eliciting Canadian population preferences for health states using the Classification and Measurement System of Functional Health (CLAMES). Chronic Dis Canada.

[21] Haagsma JA, Polinder S, Cassini A, Colzani E, Havelaar AH (2014). Review of disability weight studies: comparison of methodological choices and values. Pop Health Metrics.

[22] Gosselin R, Ozgediz D, Poenaru D (2013). A Square Peg in a Round Hole? Challenges with DALY-based “Burden of Disease” Calculations in Surgery and a Call for Alternative Metrics. World J Surg.

[23] McCord C, Chowdhury Q (2003). A cost effective small hospital in Bangladesh: what it can mean for emergency obstetric care. Int J Gynaecol Obs.

[24] Gosselin R, Thind A, Bellardinelli A (2006). Cost/DALY averted in a small hospital in Sierra Leone: what is the relative contribution of different services?. World J Surg.

[25] Gosselin R, Maldonado A, Elder G (2010). Comparative cost- effectiveness analysis of two MSF surgical trauma centers. World J Surg.

[26] Poenaru D (2013). Getting the Job Done: Analysis of the Impact and Effectiveness of the SmileTrain Program in Alleviating the Global Burden of Cleft Disease. World J Surg.

[27] Frankfurter C, Pemberton J, Cameron BH, Poenaru D (2014). Understanding the burden of surgical congenital anomalies in Kenya: an international mixed-methods approach.

[28] Kapiriri L, Frithjof NO (2002). Whose priorities count? Comparison of community-identified health problems and Burden-of-Disease-assessed health priorities in a district in Uganda. Health Expect.

[29] Brazier J (2007). Measuring and valuing health benefits for economic evaluation.

[30] Rowen D, Brazier J, Van Hout B (2015). A Comparison of Methods for Converting DCE Values onto the Full Health-Dead QALY Scale. Med Decis Making.

[31] Streiner DL, Norman GR (2008). Health measurement scales: a practical guide to their development and use.

[32] Stouthard MEA, Essink-Bot ML, Bonsle GJ (2000). Disability weights for diseases. Europ J Public Health.

[33] Essink-Bot ML, Pereira J, Packer C, Schwarzinger M, Burstrom K (2002). Cross-national comparability of burden of disease estimates: the European Disability Weights Project. Bull World Health Organ.

[34] McKenna MT, Michaud CM, Murray CJ, Marks JS (2005). Assessing the burden of disease in the United States using disability-adjusted life years. Am J Prev Med.

[35] Yoon SJ, Bae SC, Lee SI (2007). Measuring the burden of disease in Korea. J Korean Med Sci.

[36] Salomon JA, Murray CJL (2002). Estimating health state valuations using a multiple-method protocol. Summary Measures of Population Health Concepts, Ethics, Measurement and Applications.

[37] Feng Y, Devlin N, Shah K, Mulhern B, Van Hout B (2016). New methods for modelling EQ-5D-5L value sets: an application to English data. Health Economics & Decision Science (HEDS) Discussion Paper Series.

[38] Louviere JJ, Hensher DA, Swait JD (2000). Stated Choice Methods: Analysis and Applications.

[39] Schwarzinger M, Stouthard MEA, Burström K (2003). Cross-national agreement on disability weights: the European Disability Weights Project. Pop Health Metrics.

[40] Dyer SJ (2007). The value of children in African countries-Insights from studies on infertility. J Psychosomatic Obs Gynecol.

[41] Inhorn MC (2003). “The Worms Are Weak” Male Infertility and Patriarchal Paradoxes in Egypt. Men Masculinities.

[42] Magee WP, Vander Burg R, Hatcher KW (2010). Cleft lip and palate as a cost-effective health care treatment in the developing world. World J Surg.

[43] Reidpath DD (2003). Measuring health in a vacuum: examining the disability weight of the DALY. Health Policy Planning.

[44] Anand S, Hanson K (1997). Disability-adjusted life years: a critical review. J Health Econ.

[45] Arnesen T, Kapiriri L (2004). Can the value choices in DALYs influence global priority-setting?. Health Policy.

[46] King CH, Bertino AM (2008). Asymmetries of poverty: why global burden of disease valuations underestimate the burden of neglected tropical diseases. PLoS Neglected Tropical Dis.

[47] Poenaru D, Pemberton J, Frankfurter C, Cameron BH (2015). Quantifying the Disability Averted through Pediatric Surgery: a cross-sectional comparison of a pediatric surgical unit in Kenya and Canada. World J Surg.

[48] Bickler S, Ozgediz D, Gosselin R, Weiser T, Spiegel D (2010). Key concepts for estimating the burden of surgical conditions and the unmet need for surgical care. World J Surg.

