# Supplementary material for: Establishing disability weights for congenital pediatric surgical conditions: a multi-modal approach
Source: Popul Health Metr. 2017 Mar 4;15:8. doi: 10.1186/s12963-017-0125-5 (PMC5336647; doi:10.1186/s12963-017-0125-5)
Supplement: Additional file 1: — Health state information handouts. (PDF 648 kb) [file 12963_2017_125_MOESM1_ESM.pdf]

Hirschsprung's Disease 1

Background:

Hirschsprung's disease is a condition of the intestine in which the muscle cells of the bowel can't push the stool through, leading to blockage. The intestine becomes very large and may also get infected.

Children with this condition may stop having bowel movements, vomit, be very constipated, not gain weight properly, or even have severe diarrhea. These problems can happen right after birth or anytime in childhood.

The condition requires surgery, where the part of the intestine which doesn't function well is cut out. Often children also require a colostomy (an opening in their skin on their side where their stool comes out, instead of from their anus). A bag is attached (stuck) to their side around this opening to collect their stool. This bag needs to be cleaned, changed and washed regularly. Colostomies can sometimes smell bad.

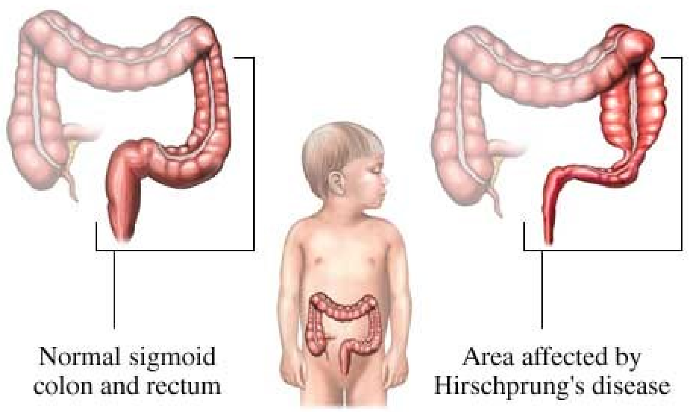

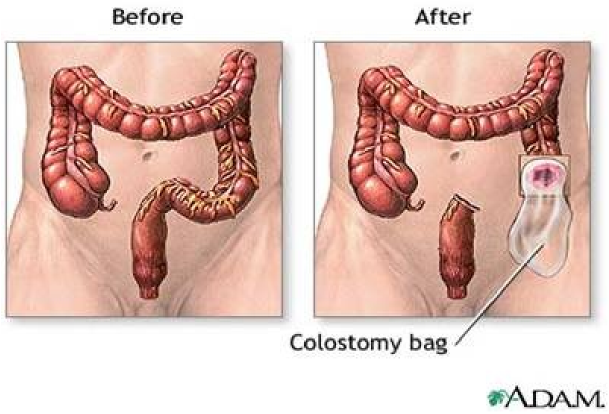

.

Hirschsprung's disease is divided in the following stages:

1) Before colostomy
2) After colostomy

We now ask you to value the following health state:

Children before colostomy: Children with Hirschsprung's disease who have not yet had a colostomy

Description:

- No problems with moving about
- No problems with washing and dressing self
- Some problems with performing usual activities
- Some / severe pain and discomfort
- Moderately anxious or depressed
- No / some social stigma
- No thinking problems
- Severe bowel problems (eg. constipation, blockage, soiling, leakage)

Hirschsprung's Disease 2

Background:

Hirschsprung's disease is a condition of the intestine in which the muscle cells of the bowel can't push the stool through, leading to blockage. The intestine becomes very large and may also get infected.

Children with this condition may stop having bowel movements, vomit, be very constipated, not gain weight properly, or even have severe diarrhea. These problems can happen right after birth or anytime in childhood.

The condition requires surgery, where the part of the intestine which doesn't function well is cut out. Often children also require a colostomy (an opening in their skin on their side where their stool comes out, instead of from their anus). A bag is attached (stuck) to their side around this opening to collect their stool. This bag needs to be cleaned, changed and washed regularly. Colostomies can sometimes smell bad.

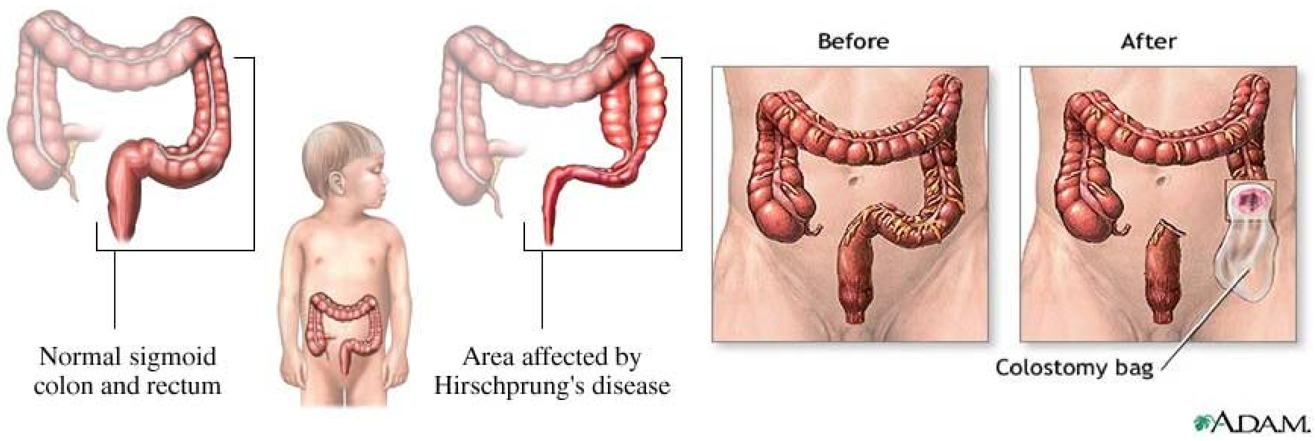

.

We now ask you to value the following health state:

Children with Hirschsprung's Disease **after** colostomy

Description:

- No /some problems with moving about
- No / some problems with washing and dressing self
- Some problems with performing usual activities (if soiling)
- Some pain and discomfort
- Some anxiety or depression
- Severe social stigma
- No thinking problems
- Some bowel problems

Imperforate anus (anorectal malformation, ARM) 1

Background:
Imperforate anus is a condition from birth in which the opening for the stool is either in the wrong place or is completely closed. In girls the opening may be very close to the vagina or even hidden inside, while in boys the opening may be on the skin behind the penis or hidden inside. As long as there is still an opening for the stool to the outside the child may live, though be leaking stool or be very constipated; without an outside opening the child would die without immediate surgery.

Surgery is required by all children with this condition. If the stool opening does exist on the skin, only one relatively small surgery is required. Otherwise children usually need 3 surgeries – a first, urgent surgery to bring the intestine to the skin (called a colostomy), followed by the main surgery to create the proper opening for the stool, then the final surgery to close the colostomy and restore the proper stool passage.

After the final surgery children may still have difficulties with stool passage. Those with milder condition may suffer from constipation, while those with the more severe condition may suffer from stool leakage all their life.

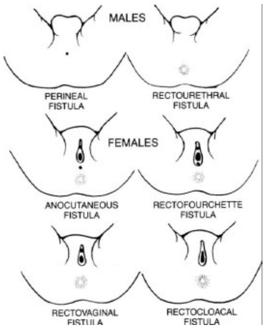

Imperforate Anus is divided in the following types:

3) Mild Imperforate Anus: No colostomy required
4) Severe Imperforate: 3 surgeries required, including a colostomy

We now ask you to value the following health state:

Children with **mild** Imperforate Anus:
Children with Imperforate Anus who only require one relatively small surgery and no colostomy

Description:

- No problems with moving about
- No problems with washing and dressing self
- Some problems with performing usual activities (because of stool leakage)
- Some pain and discomfort
- Some anxiety or depression
- Some social stigma
- No thinking problems
- Moderate bowel problems (eg. Constipation, soiling, leakage)

Imperforate Anus (anorectal malformation, ARM) 2

Background:
Imperforate anus is a condition from birth in which the opening for the stool is either in the wrong place or is completely closed. In girls the opening may be very close to the vagina or even hidden inside, while in boys the opening may be on the skin behind the penis or hidden inside. As long as there is still an opening for the stool to the outside the child may live, though be leaking stool or be very constipated; without an outside opening the child would die without immediate surgery.

Surgery is required by all children with this condition. If the stool opening does exist on the skin, only one relatively small surgery is required. Otherwise children usually need 3 surgeries – a first, urgent surgery to bring the intestine to the skin (called a colostomy), followed by the main surgery to create the proper opening for the stool, then the final surgery to close the colostomy and restore the proper stool passage.

After the final surgery children may still have difficulties with stool passage. Those with milder condition may suffer from constipation, while those with the more severe condition may suffer from stool leakage all their life.

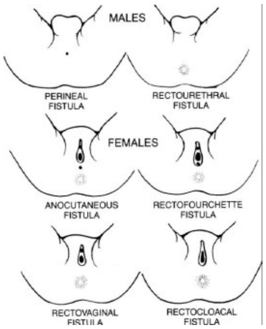

Imperforate Anus is divided in the following stages:

1) Mild Imperforate Anus: No colostomy required
2) Severe Imperforate: 3 surgeries required, including a colostomy

We now ask you to value the following health state:

Children with **Severe** Imperforate Anus:
Children with Imperforate Anus who undergo 3 surgeries – a first, urgent surgery to bring the intestine to the skin (colostomy), followed by the main surgery to create the proper opening for the stool, then the final surgery to close the colostomy and restore the proper stool passage.

Description:

- No problems with moving about
- Some problems with washing and dressing self
- Some problems with performing usual activities (because of colostomy)
- Some or severe pain and discomfort
- Some anxiety or depression
- Some social stigma
- No thinking problems
- Moderate to severe bowel problems (e.g. constipation, blockage, soiling, leakage)

Cleft lip / palate 1

Cleft lip is a separation of the upper lip, either on one side or on both sides. It is sometimes only a small separation or may go all the way into the nose. Cleft palate is a separation in the tissues that make the roof of the mouth on the inside, above the tongue. It can also be either a small or a complete separation. Each of them can happen alone or together with the other one – some children have only cleft lip, others only cleft palate, and others have both.

Children with only a cleft lip problem will look disfigured and may have problems in life with their teeth. Children who also have a cleft palate may have feeding problems as babies, then will have serious problems with speech, hearing and teeth later on.

All children with cleft lip and palate need surgery. Some will be fine after one surgery, but most will need more surgeries to fix not only the lip or palate, but also the teeth, nose, and ears.

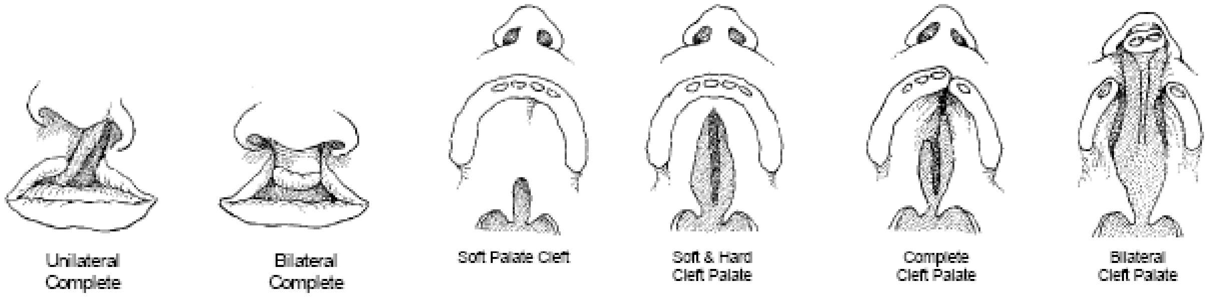

Cleft lip / palate is divided into the following states:

1. Cleft lip without cleft palate
2. Cleft palate with or without cleft lip

We now ask you to value the following health state:

Children with cleft lip **without** cleft palate

Description:

- No problems with moving about
- No problems with washing and dressing self
- Mild problems with performing usual activities (eating)
- No pain and discomfort
- Mild or severe anxiety or depression
- Severe social stigma
- No thinking problems
- No bowel/urine problems

Cleft lip / palate 2

Cleft lip is a separation of the upper lip, either on one side or on both sides. It is sometimes only a small separation or may go all the way into the nose. Cleft palate is a separation in the tissues that make the roof of the mouth on the inside, above the tongue. It can also be either a small or a complete separation. Each of them can happen alone or together with the other one – some children have only cleft lip, others only cleft palate, and others have both.

Children with only a cleft lip problem will look disfigured and may have problems in life with their teeth. Children who also have a cleft palate may have feeding problems as babies, then will have serious problems with speech, hearing and teeth later on.

All children with cleft lip and palate need surgery. Some will be fine after one surgery, but most will need more surgeries to fix not only the lip or palate, but also the teeth, nose, and ears.

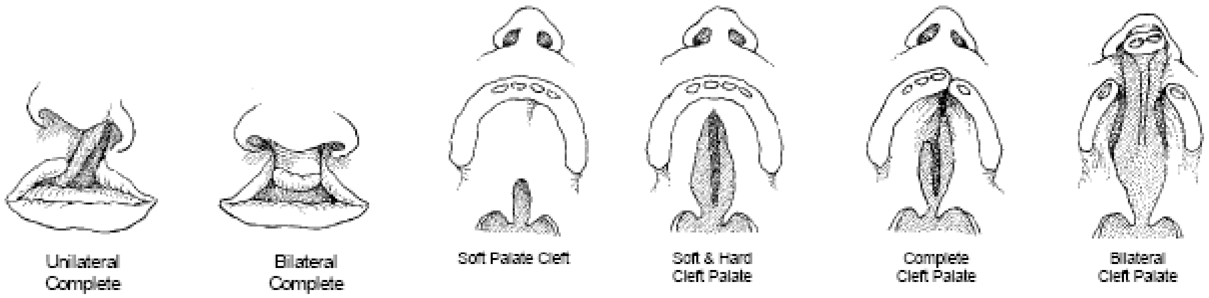

Cleft lip / palate is divided into the following states:

3. Cleft lip without cleft palate
4. Cleft palate with or without cleft lip

We now ask you to value the following health state:

Children with cleft palate, with or without cleft lip

Description:

- No problems with moving about
- No problems with washing and dressing self
- Some or severe problems with performing usual activities (eating)
- Some pain and discomfort
- Mild or severe anxiety or depression
- Some or severe social stigma
- No thinking impairment
- No bowel/urine problems

Hydrocephalus

Hydrocephalus is a condition where fluid (like water) increases in the spaces inside the brain, making the brain and the head larger. The fluid is made and drained all the time in those normal spaces, but if there is a blockage to the natural drainage then the fluid keeps increasing and the brain swells up. The blockage can happen from a problem at birth in the brain, or soon afterwards because of an infection. The pressure that builds up in the head will usually cause brain damage, which if left too long may not get better after surgery.

Parents of children with hydrocephalus will notice their child's head increasing, the soft spot on top of the head becoming hard, the eyes looking always down, and the child becoming very often upset and crying. Older children may have headaches, throwing up, feeling sleepy, and poor performance.

The child with hydrocephalus needs urgent surgery to relieve the pressure in the head. This can be done with a tube draining the fluid from the head into the child's abdomen (stomach), or using keyhole (laparoscopic) surgery.

After the surgery the head will stop growing and the child may improve, although often if the pressure has been kept for too long the damage will also remain for life. Problems such as infection or blockage of the tube can also happen.

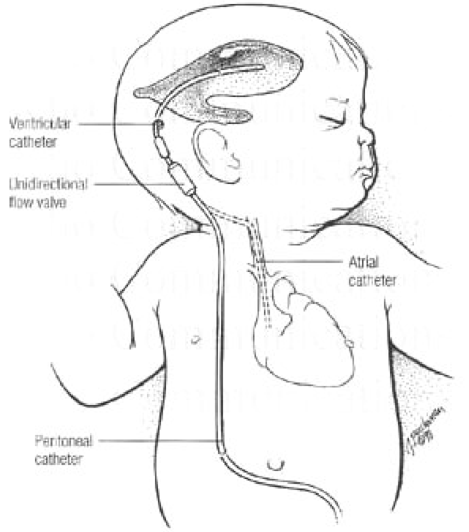

We now ask you to value the following health state:

Children with hydrocephalus

Description:

- Some / severe problems with moving about
- Some / severe problems with washing and dressing self
- Some / severe problems with performing usual activities (eg. study, family or leisure activities)
- Some pain and discomfort
- Some anxiety or depression
- Some/severe social stigma
- Some / severe thinking problems (memory, concentration, speaking, intelligence)
- No/some bowel problems (soiling, leakage)

Hypospadias 1

Hypospadias is a condition in boys where the opening for the urine is not at the tip of the penis. The opening may be along the penis, or even on the scrotum (bag) or between the legs. The penis itself may be bent downwards. Children are born that way, although if the child is not circumcised the condition may not be discovered right away.

A child with hypospadias will not be able to pee forward normally and as he grows he will often be mocked because of that. If the hole is far away from the tip of the penis, later on the man will not be able to pass his semen naturally to the woman.

Hypospadias is repaired through surgery, although sometimes – especially for those where the hole is far from the tip of the penis – it requires two or more surgeries. After successful surgery, the person can expect to function normally.

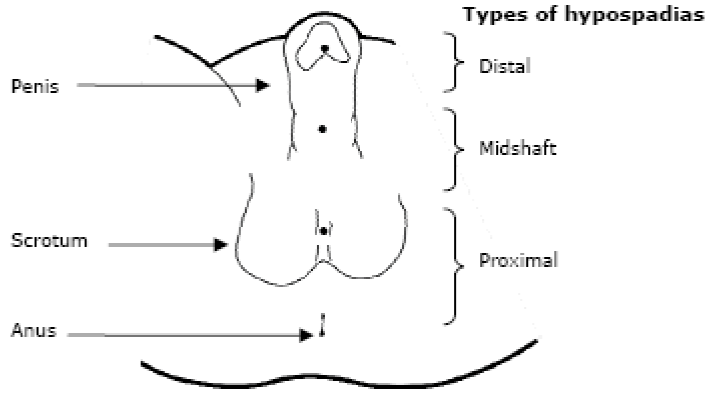

Hypospadias includes 2 states:

1. Mild hypospadias – where the hole is close to the tip of the penis (called *distal*)
2. Severe hypospadias – where the hole is far from the tip of the penis (called *proximal*)

We now ask you to value the following health state:

Children with **mild** hypospadias – children with hypospadias where the hole is close to the tip of the penis

Description:

- No problems with moving about
- No problems with washing and dressing self
- No problems with performing usual activities
- No pain and discomfort
- Some anxiety or depression
- Some social stigma
- No thinking problems
- Mild urine problems, no bowel problems

Hypospadias 2

Hypospadias is a condition in boys where the opening for the urine is not at the tip of the penis. The opening may be along the penis, or even on the scrotum (bag) or between the legs. The penis itself may be bent downwards. Children are born that way, although if the child is not circumcised the condition may not be discovered right away.

A child with hypospadias will not be able to pee forward normally and as he grows he will often be mocked because of that. If the hole is far away from the tip of the penis, later on the man will not be able to pass his semen naturally to the woman.

Hypospadias is repaired through surgery, although sometimes – especially for those where the hole is far from the tip of the penis – it requires two or more surgeries. After successful surgery, the person can expect to function normally.

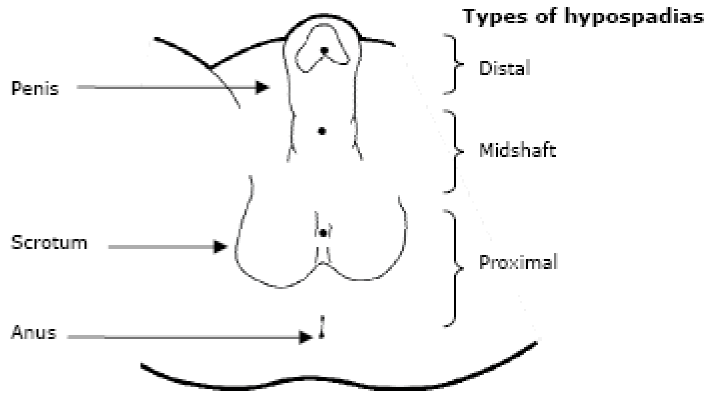

Hypospadias includes 2 states:

3. Mild hypospadias – where the hole is close to the tip of the penis (called *distal*)
4. Severe hypospadias – where the hole is far from the tip of the penis (called *proximal*)

We now ask you to value the following health state:

Children with **severe** hypospadias – children with hypospadias where the hole is far away from the tip of the penis

Description:

- No problems with moving about
- No problems with washing and dressing self
- Some problems with performing usual activities (passing urine, having sex, making children)
- No pain and discomfort
- Some anxiety or depression
- Some/severe social stigma
- No thinking problems
- Mild / severe urine problems, no bowel problems

Spina bifida 1

In this condition the spinal cord of the baby, which carries all its nerves from the brain to the body, has a defect and comes out from the backbone and through the skin. The spot where this happens can be quite low, close to the buttocks, or higher up towards the rib cage. The skin is usually open over the spot, or it may cover it; a fatty lump may also be found there.

Children born with this condition often have weakness in the legs or the legs may be completely They may also have problems with leakage of stool and urine, and may have fluid (water) building up and swelling the brain (hydrocephalus).

Surgery is needed usually right after birth to close the opening on the spine. This surgery will not make the weakness of the child better, but will help against infection. Another surgery to fix the water in the brain (hydrocephalus) is often needed soon afterwards. Whatever problems with stool or urine leakage the child had will continue after the surgery, and will need to be treated, sometimes with surgery. The children often have urine infections, which may damage the kidneys. These children also often develop a crooked spine, and may need surgery for that as well. Some children end up walking with or without help, others will need wheelchairs.

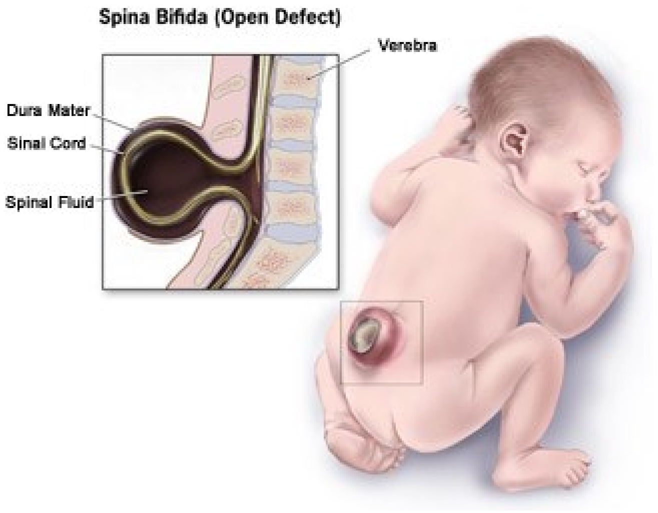

Spina bifida comes in 2 states:

1. Mild Spina bifida – with no weakness in legs
2. Severe spina bifida – with weakness in legs

We now ask you to value the following health state:

Children with **mild** spina bifida – children with spina bifida with no weakness in the legs

Description:

- No problems with moving about
- No problems with washing and dressing self
- No or mild problems with performing usual activities (because of urine/stool leakage)
- No pain and discomfort
- Some anxiety or depression
- Some social stigma
- No or mild thinking problems (if also hydrocephalus)
- No or mild bowel and urine problems (if stool/urine leakage)

Spina bifida 2

In this condition the spinal cord of the baby, which carries all its nerves from the brain to the body, has a defect and comes out from the backbone and through the skin. The spot where this happens can be quite low, close to the buttocks, or higher up towards the rib cage. The skin is usually open over the spot, or it may cover it; a fatty lump may also be found there.

Children born with this condition often have weakness in the legs or the legs may be completely They may also have problems with leakage of stool and urine, and may have fluid (water) building up and swelling the brain (hydrocephalus).

Surgery is needed usually right after birth to close the opening on the spine. This surgery will not make the weakness of the child better, but will help against infection. Another surgery to fix the water in the brain (hydrocephalus) is often needed soon afterwards. Whatever problems with stool or urine leakage the child had will continue after the surgery, and will need to be treated, sometimes with surgery. The children often have urine infections, which may damage the kidneys. These children also often develop a crooked spine, and may need surgery for that as well. Some children end up walking with or without help, others will need wheelchairs.

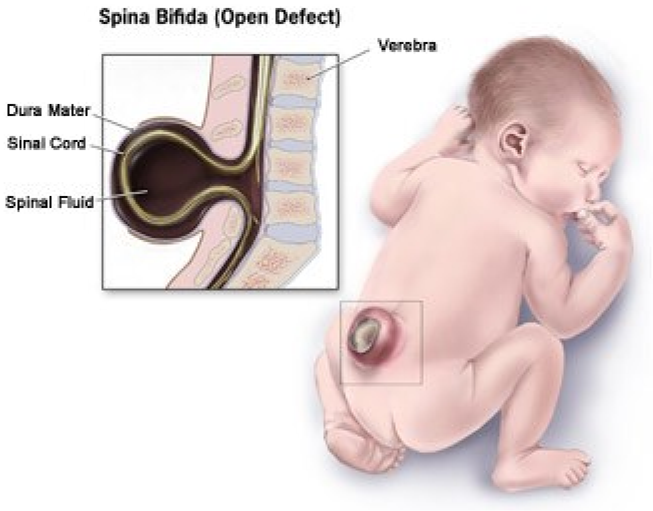

Spina bifida comes in 2 states:

3. Mild Spina bifida – with no weakness in legs
4. Severe spina bifida – with weakness in legs

We now ask you to value the following health state:

Children with **severe** spina bifida – children with spina bifida with weakness in the legs

Description:

- Severe problems with moving about
- Some problems with washing and dressing self
- Severe problems with performing usual activities
- No or some pain and discomfort
- Some or severe anxiety or depression
- Severe social stigma
- No or some thinking problems (if also hydrocephalus)
- Severe bowel and urine problems (stool/urine leakage)

Undescended testes

In this condition the boy is born with one, or both, testicles not found in the usual place (the scrotum). If nothing is done about that, the boy is at increased risk of not being able to bear children, and also at higher risk of having cancer of the testicle later in life.

The condition can be fixed through a relatively small surgery in which the testicle is found and brought down into the scrotum. If this is done at an early age usually there are no more problems later on in life, although the child will need to be checked regularly afterwards.

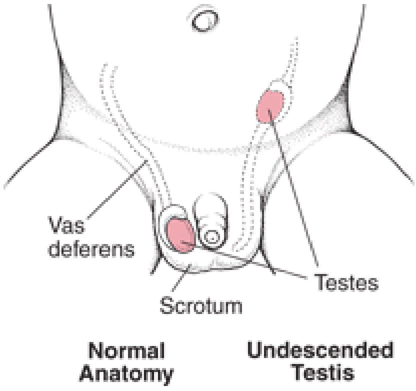

We now ask you to value the following health state:

Children with undescended testes

Description:

- No problems with moving about
- No problems with washing and dressing self
- No or some problems with performing usual activities (because of possibly not being able to have children)
- No pain and discomfort
- Some anxiety or depression (because of risk of cancer and not having children)
- No or some social stigma
- No thinking problems
- No bowel or urine problems

Abdominal wall defects (gastroschisis / omphalocele) 1

In these 2 conditions the abdomen (stomach) of the baby is opened from birth and some of the intestines (and sometimes liver) are outside – either uncovered or under a thin layer of tissue. The hole may be small or large, and the organs outside may be small or large. The babies may have other problems from birth as well, including blockage of the intestines.

Without surgery or other treatment all these babies would die from infection. The surgery must be done urgently to restore the organs inside and close the wall of the abdomen. If the organs outside are very large they are first placed in a special bag for a few days to let the abdomen stretch slowly, or the tissue on top is kept free of infection until the skin covers the organs. In that case surgery is still needed later on when the child is older, to close the muscles properly. If the intestines are also blocked the child will often need another surgery to fix that.

After the surgeries are done the child will usually be well, although its intestines may not work for several weeks and even months.

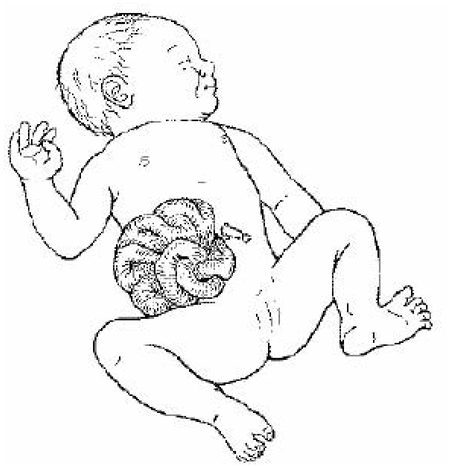

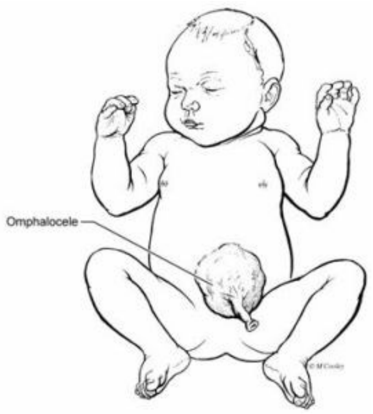

Abdominal wall defects are divided in 2 states:

1. Mild abdominal wall defects – where the hole is small and not much of the bowel is outside;
2. Severe abdominal wall defects – where the hole is large and/or much of the bowel and other organs are outside

We now ask you to value the following health state:

Children with **mild** abdominal wall defects –
children with abdominal wall defects where the hole is small and not much of the bowel is outside

Description:

- No problems with moving about
- No problems with washing and dressing self
- No / some problems with performing usual activities
- Some pain and discomfort
- Some anxiety or depression
- Some social stigma
- No thinking problems
- No or some bowel problems (if bowel blockage)

Abdominal wall defects (gastroschisis / omphalocele) 1

In these 2 conditions the abdomen (stomach) of the baby is opened from birth and some of the intestines (and sometimes liver) are outside – either uncovered or under a thin layer of tissue. The hole may be small or large, and the organs outside may be small or large. The babies may have other problems from birth as well, including blockage of the intestines.

Without surgery or other treatment all these babies would die from infection. The surgery must be done urgently to restore the organs inside and close the wall of the abdomen. If the organs outside are very large they are first placed in a special bag for a few days to let the abdomen stretch slowly, or the tissue on top is kept free of infection until the skin covers the organs. In that case surgery is still needed later on when the child is older, to close the muscles properly. If the intestines are also blocked the child will often need another surgery to fix that.

After the surgeries are done the child will usually be well, although its intestines may not work for several weeks and even months.

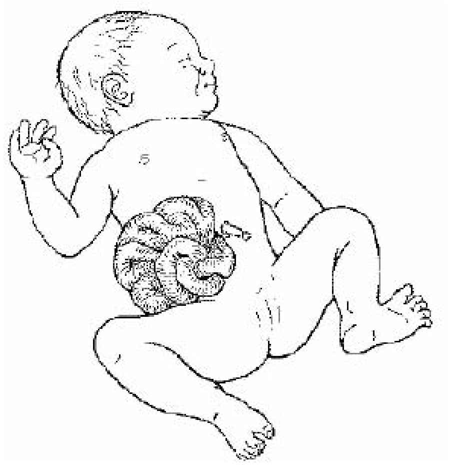

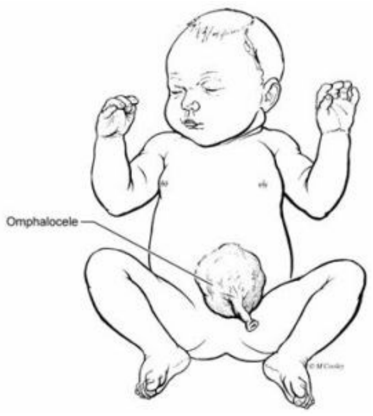

Abdominal wall defects are divided in 2 states:

3. Mild abdominal wall defects – where the hole is small and not much of the bowel is outside;
4. Severe abdominal wall defects – where the hole is large and/or much of the bowel and other organs are outside

We now ask you to value the following health state:

Children with **severe** abdominal wall defects –
children with abdominal wall defects where the hole is large and / or much of the bowel and other organs are outside

Description:

- Some problems with moving about
- Some problems with washing and dressing self
- Some / severe problems with performing usual activities
- Some / severe pain and discomfort
- Some anxiety or depression
- Some / severe social stigma
- No thinking problems
- Some / severe bowel problems (bowel blockage or very slow bowel)

Intestinal atresia

In this condition the intestines of the baby are blocked from birth. The baby usually cannot pass stool, its abdomen (stomach) gets swollen, and the baby vomits any food given.

Surgery is required right away for these babies, as they cannot live with the blockage. In most cases one surgery only is required to fix the problem, after which the babies can eat again. In some cases however the intestines continue not to function well or are too short, and the baby needs nutrition through the veins for weeks or even months.

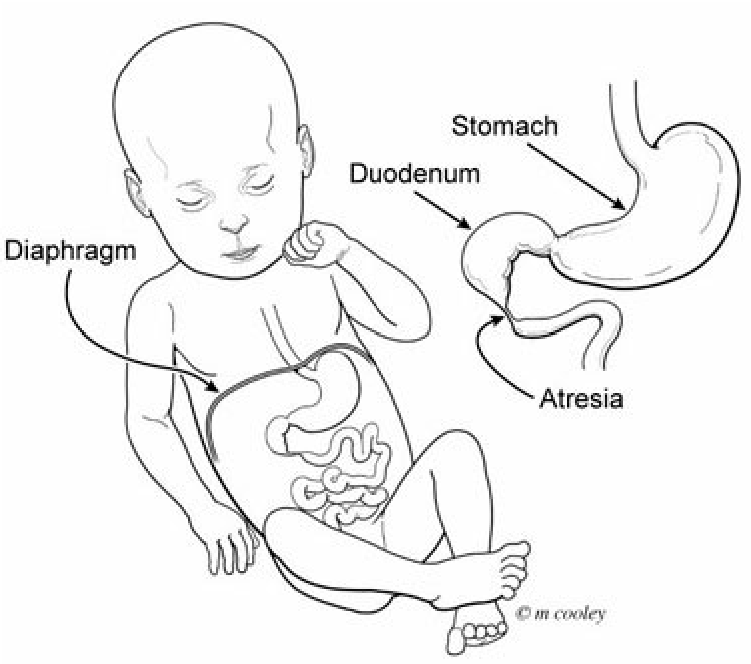

We now ask you to value the following health state:

Children with intestinal atresia

Description:

- No problems with moving about
- No problems with washing and dressing self
- Some problems with performing usual activities (eating)
- Severe pain and discomfort
- Severe anxiety or depression
- Some social stigma
- No thinking problems
- Severe bowel problems (complete blockage)
